# Barriers and Facilitators Associated with Physical Activity in the Middle East and North Africa Region: A Systematic Overview

**DOI:** 10.3390/ijerph18041647

**Published:** 2021-02-09

**Authors:** Sonia Chaabane, Karima Chaabna, Sathyanarayanan Doraiswamy, Ravinder Mamtani, Sohaila Cheema

**Affiliations:** Institute for Population Health, Weill Cornell Medicine—Qatar, Education City, Doha 24144, Qatar; sonia.chaabane.phd@gmail.com (S.C.); kac2047@qatar-med.cornell.edu (K.C.); sdo4003@qatar-med.cornell.edu (S.D.); ram2026@qatar-med.cornell.edu (R.M.)

**Keywords:** physical activity, barriers, inactivity, systematic review, Middle East, North Africa

## Abstract

Increasing physical inactivity levels in the Middle East and North Africa (MENA) region is a public health concern. We aimed to synthesize barriers and facilitators to physical activity and make appropriate recommendations to address physical inactivity. We conducted an overview of systematic reviews on physical activity barriers and facilitators in 20 MENA countries by systematically searching MEDLINE/PubMed and Google Scholar for systematic reviews published between 2008 and 2020. Our overview included four systematic reviews and 119 primary studies with data from 17 MENA countries. Lack of suitable sports facilities, time, social support and motivation, gender and cultural norms, harsh weather, and hot climate were the most commonly reported barriers to physical activity. Socio-demographic factors negatively associated with physical activity participation include advanced age, being female, less educated, and being married. Motivation to gain health benefits, losing/maintaining weight, being male, dietary habits, recreation, and increased Body Mass Index are positively associated with increased levels of physical activity. Interventions promoting physical activity in MENA should target schoolchildren, women and girls, working parents, and the elderly. Country-specific sociocultural and environmental factors influencing physical activity should be considered in the design of interventions. Current and future policies and national interventions must be consistently evaluated for effectiveness and desired outcomes.

## 1. Introduction

For optimal health benefit, it is recommended for adults to accumulate at least 150 min of moderate-intensity physical activity (PA) per week [1]; whereas, at least 60 min per day of moderate-to-vigorous-intensity PA helps children and youth maintain a healthy cardiorespiratory and metabolic risk profile [2]. Within the Middle East and North Africa (MENA) region, it is estimated that about 49% of adults and 75% of the youth population are not sufficiently active to meet the recommended international guidelines for PA [3].

Countries in the MENA region have some of the highest rates of diabetes and obesity in the world [4,5,6]. In recent decades, urbanization and advances in technology and transportation have led to increased sedentary lifestyles in the region [7]. Changes in the working environment (working from home, widespread use of telecommunication, etc.) have increased the time that individuals spend sitting [8]. The reduction in daily energy spent at work led to an increased body weight [8]. Moreover, low PA participation is of concern because it may have a detrimental effect on mental health and quality of life for children, youth, and adults [2,9]. The current living environment in several MENA countries is characterized by an increased availability of unhealthy food combined with a lifestyle requiring low levels of PA [7], promoting high energy intake, and low energy expenditure [7,10,11,12,13], all of which are major risk factors for non-communicable diseases (NCDs). Reducing NCD risk has become an important goal for the whole region due to the changing demographics in low- and middle-income countries in the region and high health care and treatment costs associated with these diseases [14].

Although some MENA countries are increasing the number of parks available and improving access to sports facilities for their residents [15,16], others are lagging behind. In order for MENA countries to be successful in implementing interventions that facilitate PA, it is important to understand the barriers and facilitators of PA programs within their specific populations. We need to identify country-specific factors associated with low PA participation in order to better customize interventions to local needs [7]. Our study objective is to identify and explore factors positively associated (facilitators) and negatively associated (barriers) with PA. This review will help inform academics, researchers, and policymakers, thus enabling them to make informed decisions about how to improve PA participation in the MENA region.

## 2. Materials and Methods

We conducted a systematic overview of published systematic reviews (SRs) on the epidemiology of PA in the MENA region. In a previous publication, we synthesized data on the prevalence of PA and sedentary behavior [3]. In this present study, we focus on synthesizing country-specific barriers and facilitators associated with PA in the MENA region.

### 2.1. Search Strategy and Selection Criteria

A broad search strategy was developed to systematically identify any type of review on all health issues in any MENA country [3,17]. We used search terms related to the names of MENA countries, regions and sub-regions. The detailed search strategy is available as Supplementary Materials (Panel 1). No restrictions to a specific health condition or language of publication were applied at this stage [18]. The detailed search strategy is available in our previous publications [3,17]. Two independent reviewers systematically searched the Medical Literature Analysis and Retrieval System Online (MEDLINE) through the search engine PubMed. We included publications since 2008—the publication year of the first version of the Cochrane Handbook for Systematic Reviews of Interventions [19]—up to November 2019. We also searched Google Scholar for primary studies (grey and non-grey literature), with no date nor language restriction, up to April 2020. Additionally, a manual search of the references from the included studies was also conducted.

### 2.2. Inclusion and Exclusion Criteria

While we searched for any type of literature review, we included only SRs. An SR was defined as a literature review that explicitly used a systematic literature search of at least one electronic database to identify all studies that met pre-defined eligibility criteria, and that reported the process of study selection [19]. We considered any kind of PA that people do as part of their everyday lives (leisure time PA and/or physical labor).

MENA countries, where the primary official languages and/or the medium of instruction in the colleges/universities was Arabic, English, French, and/or Urdu, were included. The authors of this overview are fluent and proficient in these languages [17]. The 20 MENA countries included in the study are Algeria, Bahrain, Djibouti, Egypt, Iraq, Jordan, Kuwait, Lebanon, Libya, Morocco, Oman, Pakistan, Palestine, Qatar, Saudi Arabia, Sudan, Syria, Tunisia, the United Arab Emirates (UAE), and Yemen.

### 2.3. Data Screening

The retrieved reviews were downloaded into Endnote (version X8.2) and duplicates were removed. Using Rayyan software [20], two independent reviewers conducted the multi-stage screening. Discrepancies in the inclusion of SRs were resolved through discussion with a third reviewer.

### 2.4. Data Extraction

One reviewer extracted the data, and another reviewer checked the accuracy of the extracted data. From each included SR, the following characteristics were extracted: reported factors associated with PA, study period, geographical coverage of the literature search, the corresponding MENA countries for which data was available, and literature sources, as well as the number of included studies and the study target population. The characteristics of the primary studies included in our overview, along with the methodological quality assessment and study selection process, are described in a previous publication [3]. Where available, we extracted factors associated with PA specific to different age groups (youth and adults) and gender (boys/girls or males/females).

We classified the MENA countries into high and low-middle income countries using the World Bank classification by income [21]. High-income MENA countries include Bahrain, Kuwait, Oman, Qatar, Saudi Arabia, and UAE [21]. Low- and middle-income MENA countries include: Algeria, Djibouti, Egypt, Iraq, Jordan, Lebanon, Libya, Morocco, Pakistan, Palestine, Sudan, Syria, Tunisia, and Yemen [21]. We used the number of studies reporting each barrier, facilitator, or correlate of PA in each country to identify the most frequently reported barrier, facilitator, and correlate of PA in those countries.

### 2.5. Methodological Quality Assessment

The original AMSTAR critical appraisal tool for SRs [22] was used by two independent reviewers to perform and discuss the quality assessment of the included SRs.

### 2.6. Data Synthesis

For the purpose of data synthesis and discussion, reported factors associated with PA were first grouped into six categories: (1) sociodemographic, (2) intrapersonal, (3) interpersonal, (4) physical environment, (5) sociocultural, and (6) organizational and policy [23]. We further sub-grouped these factors as barriers (factors negatively associated with PA) and facilitators (factors positively associated with PA) among the whole population of any age group residing in a MENA country.

## 3. Results

**Figure 1:**
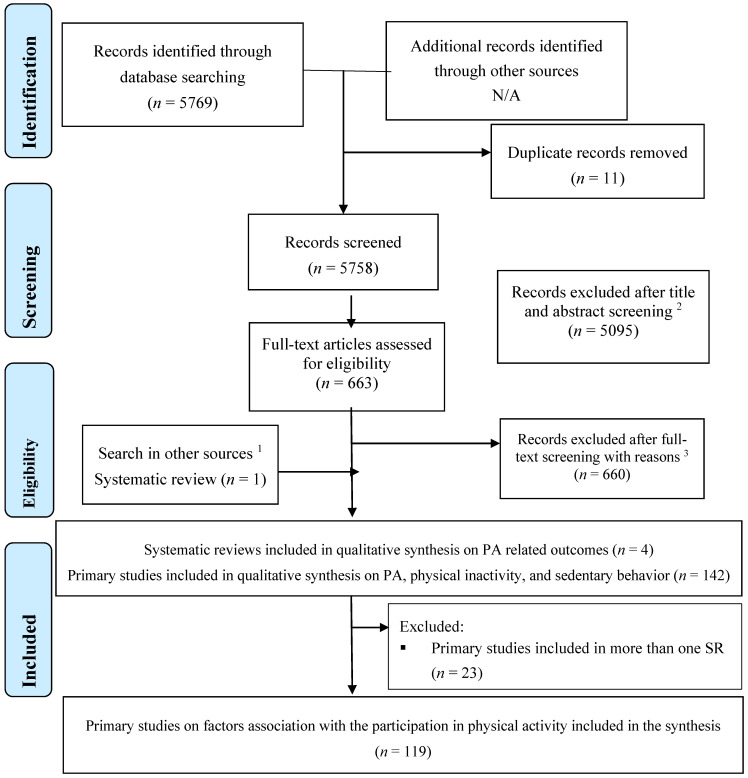
Preferred Reporting Items for Systematic Reviews and Meta-Analyses (PRISMA) 2009 flowchart of the systematic reviews inclusion. ^1^ Google Scholar and manual search of the references in the included studies. ^2^ Systematic reviews were excluded from the overview because they did not meet our inclusion criteria; ^3^ not a systematic review (*n* = 116), no data on barriers to physical activity (*n* = 507), not a population from MENA (*n* = 34), duplicate (*n =* 1), and publication in Portuguese (*n* = 1), a systematic review with qualitative data only (*n =* 1).

Our search strategy identified four SRs that included 142 primary studies reporting on factors (barriers or facilitators) associated with PA in at least one of the 20 MENA countries (Figure 1). After excluding primary studies included in more than one SR (*n* = 23), we considered a total of 119 studies for the qualitative synthesis. 

### 3.1. Characteristics and Methodological Quality of the Included Systematic Reviews

The four SRs included in our overview are described in Table 1. Data on barriers and facilitators of PA were identified for seventeen MENA countries, namely Bahrain, Egypt, Jordan, Iraq, Kuwait, Libya, Lebanon, Morocco, Oman, Pakistan, Palestine, Qatar, Saudi Arabia, Syria, Sudan, Tunisia, and the United Arab Emirates (UAE). Data on children and adolescents (youth), as well as adults, were identified. No SR reported the list of excluded studies, assessed publication bias, included the funding source of both the SR and of the included primary studies, nor searched for grey literature sources as per the AMSTAR recommendations [24].

### 3.2. Synthesis of Data on PA Barriers and Facilitators

Table 2 describes extracted barriers and facilitators associated with PA in MENA countries. Two studies reported data on the sub-region of Arab countries, namely Algeria, Djibouti, Egypt, Jordan, Kuwait, Libya, Morocco, Oman, Palestine, Syria, and the UAE. Most studies reporting PA barriers and facilitators were conducted in high-income MENA countries, mainly Saudi Arabia and the UAE. Except for the SR of Benjamin, 2013 [28], all SRs included data on adults and youth. Only the SR of Al-Hazzaa, 2018 [26], segregated PA barriers and facilitators by gender and age groups (children, adolescents, and adults) among the Saudi population.

Considering any population (all ages, both genders), the most commonly reported barriers to PA were: lack of suitable sports facilities (number of studies reporting this factor [*n*] = 43), lack of time (*n* = 36), gender norms (e.g., difficulty for women in joining a gym) and cultural norms (e.g., norms promoting overeating) (*n* = 32), harsh weather and hot climate (*n* = 24), lack of social support (*n* = 19), and lack of motivation (*n* = 15). These barriers were reported in most of the MENA countries and more frequently in the high-income countries. The most commonly reported sociodemographic barriers to PA were age (elderly people being more inactive than younger people (*n* = 25), being less educated (*n* = 15), being married (*n* = 7), and being female (*n* = 5). Considering any population (all ages, both genders), the commonly reported PA facilitators were being male (*n* = 7), motivation to gain health benefits (*n* = 7), losing/maintaining weight (*n* = 6), the consumption of foods high in fats/salt/sugar (*n* = 5), increased Body Mass Index and waist circumference (*n* = 4), recreation (*n* = 4), and consumption of healthy food (fruits, vegetables, milk) (*n* = 3).

PA barriers for boys (children and adolescents) included obesity (*n* = 2), age (*n* = 1), inadequate school physical education program (*n* = 1), lack of sports facilities (*n* = 1), lack of time (*n* = 1), and lack of friends’ support (*n* = 1). No data on PA barriers among girls (children and adolescents) was reported in the included SRs. PA barriers among adult males included lack of time (*n* = 2), no suitable place (*n* = 1), being married (*n* = 1), work in private section (*n* = 1), less educated (*n* = 1), and high educational level (*n* = 1). PA barriers for adult females included lack of time (*n* = 3), no suitable place (*n* = 1), no one to exercise with (*n* = 1), no access to sport facilities (*n* = 2), low self-efficacy (*n* = 1), working in private sector (*n* = 1), working seven or more hours (*n* = 1), and age (*n* = 1). PA barriers for youth included high screen time (*n* = 1), being female (*n* = 4), obesity (*n* = 2), older age (*n* = 4), inadequate school physical education program (*n* = 1), knowledge about obesity prevention (*n* = 1), lack of time (*n* = 3), no suitable place (*n* = 1), lack of sports facilities (*n* = 1), lack of friends’ support (*n* = 1), waist circumference (*n* = 1), sleeping hours (*n* = 1), and eating habits (*n* = 1).

PA facilitators among boys (children and adolescents) included enhancing muscle and strength (*n* = 1), enjoyment (*n* = 1), and improving body appearance (*n* = 1). No data were available for specific PA facilitators among girls in the included SRs. Male PA facilitators included age (*n* = 2), getting health benefits (*n* = 2), recreation (*n* = 2), socializing (*n* = 1), being married (*n* = 1), and losing weight (*n* = 1). Female PA facilitators included getting health benefits (*n* = 4), lose/maintain weight (n = 4), have fun (*n* = 1), enjoyment (*n* = 1), being married (*n* = 1), and recreation (*n* = 1). PA facilitators among children and adolescents included getting health benefits (*n* = 1), lose weight (*n* = 1), recreation (*n* = 1), socializing with others (*n* = 1), enhancing muscle and strength (*n* = 1), enjoyment (*n* = 1), and improving body appearance (*n* = 1).

## 4. Discussion

Our overview included four SRs and 119 primary studies, with data from 17 MENA countries. The lack of suitable sports facilities, time, social support and motivation, gender and cultural norms, and harsh weather and hot climate were the most commonly reported barriers to PA. Specific socio-demographic factors, such as advanced age, less education, being female, and being married, were found to be negatively associated with PA. Gaining health benefits, being male, losing/maintaining weight, dietary habits, recreation, and increased Body Mass Index were identified as PA facilitators. Most data reported on the topic were available from the high-income MENA countries.

PA is developed as a behavior through complex and dynamic interrelations between multiple factors including intrapersonal, interpersonal, and environmental [143,144]. Several of PA facilitators and barriers reported in MENA countries are also reported worldwide. Our study finds variability in the reported facilitators and barriers within the MENA countries. Borrowing from international and regional experience, while some blanket interventions would be required, there is also a need to design country-specific interventions considering local sociocultural and environmental factors influencing PA. Available evidence from low-middle income countries indicates that multicomponent (e.g., media, behavioral, social, policy, and environmental), multisector (e.g., public health, transportation, recreation, health care), and multisite (e.g., work, school, community organization) community-wide campaigns, can be effective in increasing participation in PA [144]. However, there are important barriers to policy implementation, such as insufficiently trained workforce to implement PA policies. These barriers must be overcome before progress in increasing PA can be expected [144]. An important next step would be to build capacity for physical activity surveillance, intervention research, and policy implementation, especially among low-middle income countries [144].

### 4.1. Intrapersonal Factors

The presence of a health condition, such as being overweight or having a pre-existing medical illness, as well as the consumption of foods high in fats/salt/sugar, were identified as barriers to PA. However, in some studies, they were also identified as facilitators to PA, as explained below. Although the presence of a health condition can restrict PA participation, for some individuals, the health condition can act as a ‘motivator’ to become more physically active in order to improve their health status [28]. Higher PA levels observed in some studies among youth who were consuming foods high in sugar and fast food (rich in fats and salt) can be explained by the fact that, following sporting events and environments, youth may be more likely to consume sugary beverages and unhealthy snacks (through vending machines, commercial presences, etc.) [75]. A reported rise in the consumption of sports drinks and energy drinks (generally high in sugar) [145,146], which are often marketed to youth to assist or enhance exercise or sports performance, can be linked to increased consumption among youth who follow exercise regimens [146].

Individual perception of the impact of PA on health status influences the actual practice of PA. Health benefits related to PA, such as improved balance and walking ability, reduced muscle pain, improved sleep, and muscle strengthening, were found to be major facilitators for the initiation and continuation of PA [28,147,148,149]. However, failure to perceive the health benefits has been reported as a barrier to PA [148,150].

Particularly in the elderly, inadequate levels of PA were associated with fear of injury or pain [148,151]. Health care providers must be cognizant of these fears when counseling the elderly population [148,149]. It is important to minimize the risk of injury and the fear associated with it by increasing self-awareness and choosing the correct exercise that is appropriate for the individual’s age, fitness level, skill level, and health status [152]. To prevent injury from PA, individuals should be encouraged to practice warm up and cool down activities before and after the PA session. Individuals may want to initiate their PA regimen under the supervision of an exercise instructor to ensure proper technique and to choose the appropriate program for them. Due to safety concerns, fear of walking at night outside the home has been cited as deterrent to PA [148].

Motivation for PA was associated with increased PA participation [7,153]. Motivating factors for PA include pleasure experienced while exercising [153] and scheduling PA for times in the day or the week when energy levels are high. Convincing oneself that PA increases energy levels is also a motivator for increased PA participation [152]. Preference to engage in PA as a group or while enjoying the natural scenery may also motivate others [148,150]. These motivational aspects can be taken into consideration while planning interventions to promote PA.

Lack of time was reported as a universal barrier to PA for all age groups [7,28,73,150,154]. Reasons reported for the unavailability of time for PA include household responsibilities (e.g., household chores, childcare), extra office work for men, frequent social gatherings, and time management challenges related to heavy school workload [28,148,155]. To address this barrier, one can identify at least five 30-min time slots weekly for the purpose of PA. Monitoring daily activity with the aid of a smart phone and fitness trackers, PA apps can also help encourage individuals to indulge in regular PA [156,157]. Walking or riding a bike to work, taking the stairs instead of the elevators, exercising while watching TV, and parking farther away from a destination are effective ways to add PA to a daily routine [158]. Where possible, taking advantage of work PA facilities or programs, walking while on a call, or stretching and moving around can also be useful in facilitating PA [152].

### 4.2. Interpersonal Factors

The absence of social support was reported as a barrier to PA [7,148] in high and low-middle income countries of the MENA region. This is also observed in other regions of the world [144]. It has been shown that adolescents who have inactive parents and minimal support from their friends to encourage PA tend to be physically inactive [159]. Adolescents who are appreciated by friends as being athletically competent show positive feelings towards practicing PA [7]. Having good informal (family friends) and formal (health care provider) support systems increases PA participation [28]. Social support can increase the self-confidence of individuals and motivate them to begin exercise regimens [28,148,160]. The possibility of exercising together is likely to motivate friends and family members to engage in PA [28]. Other facilitators for PA include planning social activities involving exercise and developing new friendships with physically active people by joining a gym, an exercise group, or a hiking club [152]. Elderly individuals may need more social support than younger adults to remain physically active [150]. Health professionals can encourage elderly people to participate in group exercises appropriate for their age, skill, and ability.

### 4.3. Environmental Factors

Lack of sports facilities decreases motivation for PA and was identified as one of the major barriers to PA in our study [161,162]. The lack of sports facilities, lack of support from the immediate environment (e.g., school or institution), and societal and family restriction appears to prevent young Saudi females students from participating in PA [49]. With increasing regional urbanization [163], urban design facilitating PA has been suggested as a potential strategy to mitigate the lack of PA facilities in the urban areas of low- and middle-income MENA countries [144]. With the right level of commitment and resource allocation, this strategy is likely to work and yield good results.

The costs of accessing sports facilities was reported as a barrier to PA in high-income countries of the MENA region and internationally [153]. Selecting PA activities that require minimal use of fitness/sports facilities or equipment, such as walking or jogging, and identifying inexpensive, convenient resources available in the community, such as community education programs, parks, and recreation programs, are likely to mitigate the impact of cost as a barrier to PA [28,152]. Construction of playgrounds, sidewalks, parks, cycling routes/paths, or other communal fitness facilities could motivate individuals to engage in regular PA, such as walking and cycling. Governments play an important role in supporting and funding sports facilities. An example of this is the creation of sports cities, such as the Aspire Zone in Qatar, offering several sports facilities ranging from sports venues, a sports hospital and academies, to parks and open-air activities [164]. The integration of such sports-oriented areas into urban cities demonstrates considerable improvement in PA indicators (overall PA, organized sport participation, sedentary behaviors, physical education at school, government allocation of funds and resources) [165]. In addition, the availability of resting space, such as benches for resting in between long walks, is important to ensure easy access to a safe and pleasant place for exercise [148,166].

Extreme weather (very cold or very hot seasons) was linked to a decrease in PA levels in several MENA countries and globally [73,150,153,166,167]. A higher participation in PA was observed during sunny weather and at moderate temperatures (15–27 °C) [148]. To facilitate PA in extreme weather, one can choose activities, such as indoor cycling, aerobic dance, indoor swimming, stair climbing, or mall walking, that are always available regardless of the weather [152].

### 4.4. Population Groups, Sub-Groups, and Facilitators

Youth engage in lower levels of PA compared to adults in the MENA region, as well as globally [3]. Some data indicate that midlife adults are less likely to engage in PA and are probably more at risk for unhealthy aging than young adults and the elderly [168]. Midlife adults also perceive fewer health benefits of PA than young and older adults do [168]. Decline in PA is well-documented worldwide once people attain parenthood, particularly for women [169,170,171]. Parents face numerous barriers to PA including family responsibility, guilt of not spending adequate time with the family, lack of support, scheduling constraints, and work [158]. Understanding barriers in population sub-groups is essential for developing age-appropriate interventions to promote PA. Parents who regularly engage in PA during their daily routine have developed strategies that allow them to balance household and employment responsibilities. These strategies include being active with children or during children’s activities, making time/prioritizing, considering benefits to health and family, having support available (family, friends, or child minder), and being a role model for children [158]. Working mothers have been identified as a population that could benefit significantly from interventions that are custom tailored to promote and facilitate PA among them [169,171]. Working parents can benefit from interventions that teach them strategies to overcome barriers to PA participation and to enable them to prioritize it parallel to the demands of parenthood [158].

In both adults and youth, males engage in PA more than females [3,7]. This gender difference is generally prevalent globally [172,173,174]. In MENA countries, young females as compared to young males reporting significant barriers related to lack of energy, greater interest in other activities, lack of encouragement, worries about looks, and time constraints from academic responsibilities and family obligations [7,103]. Among youth, boys place more importance on being good in sports, whereas girls focus on good grades and being attractive [175]. Attempts to regulate weight was the main motivator for meeting the recommended levels of PA among youth in Oman and in the U.S. [103,159]. Personal and social barriers to PA are higher among females than males in Arab countries [7,28,159]. These include the traditional roles for women and family obligations, lack of social support for women to exercise, and the use of housemaids [28]. In addition, greater freedom and additional facilities to engage in PA and other recreational activities were more available to males as compared to females [7,176]. Proposed interventions to increase PA participation among women included the provision of additional women only sessions or facilities to reduce anxiety related to self-consciousness [177]. Providing enhanced support to girls during the transition from secondary school to college or university or leaving school for employment where levels of sports participation may be affected could also help with their increased uptake of PA [177].

Students in some MENA countries reported limited opportunities (less support from teachers, and lack of time, family/cultural constraints) for exercising [7,103] as a barrier to PA. It is essential to maximize the role of schools in increasing the awareness about, and creating an environment that facilitates, PA. Although young adults in general seem to be aware of benefits related to PA, those from a lower socioeconomic background might have lower levels of awareness [34,49]. Obese university students perceive a higher number of barriers to practicing a healthy lifestyle, including engagement in PA, than non-obese students [73]. PA barriers in these students are related to motivation, enjoyment, and skills or ability to exercise [73].

The appeal of television, playing electronic games, and use of computers and mobile devices has increased sedentary time for children [176]. The focus of future studies should be on investigating the barriers to participation in physical education classes and exploring how these barriers can be addressed [7,103]. Inculcating PA habits among schoolchildren and adults can play an important role in the prevention and control of NCDs. Both sociocultural and intrapersonal factors influencing PA participation must be taken into account, as well as the environmental factors.

### 4.5. Strengths and Limitations

To our knowledge, this is the most comprehensive systematic overview on PA barriers and facilitators in the MENA region. The country-specific data on PA barriers will serve as a benchmark for epidemiologists and public health interventionists and can help direct future investigation and research. Limitations include the restriction of the search strategy to PubMed/MEDLINE and Google Scholar. However, the included SRs have searched several literature sources for primary studies, which minimizes the risk of publication bias in our overview. The included studies on factors associated with participation in PA were published up to 2018; new studies from MENA countries may have been published since. Limited data on organizational and policy level factors are available for the region. The absence of data on barriers and/or facilitators of PA in some MENA countries does not mean that PA barriers or facilitators are non-existent in those countries. In addition, other barriers and facilitators, not yet identified in the included studies, could also exist in the MENA countries. We identified barriers predominantly for high-income MENA countries. This could be explained by a higher number of research studies being conducted in these countries rather than the actual existence of increased barriers to PA as compared to low- and middle-income MENA countries.

The majority of the included studies were cross-sectional and had not been designed to assess causal association. SRs reporting these studies have not provided an adequate description of the type of the effect size (e.g., relative risk, odds ratio, correlation), the control for potential confounders, and the strength of the association to determine whether the identified barriers or facilitators are independently associated with the participation in PA. We considered these factors as potential barriers or facilitators based on the study’s conclusions. Therefore, further studies are needed for confirmation. The SRs identified variation in PA measurement tools and in the methodology used to conduct the studies, as well as in the methodological quality between the included studies. These limitations within the included studies may prevent deriving high quality evidence. However, the evidence that we have identified and synthesized in this overview is informative and can guide future research. Some identified barriers and facilitators in MENA countries were also identified in other countries worldwide, which support our findings.

## 5. Conclusions

Several of the reported personal and environmental barriers to PA in MENA countries are universal. The lack of suitable sports facilities and extreme weather conditions are more pronounced in some of the MENA countries than others. Advanced age and being a female, less educated, or married are associated with low PA participation. Gaining health benefits, losing/maintaining weight, dietary habits, recreation, and increased Body Mass Index were positively associated with increased levels of PA. Further research is needed to identify additional aspects of gender- and age-specific PA barriers and facilitators. Most retrieved data included on the topic in this paper was obtained from high-income MENA countries, which may have relevance in furthering our research goal of optimum physical activity for the region at large.

Interventions to promote PA in MENA countries should target schoolchildren, working parents, and the elderly. Specific attention should be paid to addressing specific barriers to PA among women and girls. Support from parents, friends, and teachers for PA must be encouraged. Programs and activities that address barriers and constraints can be expected to increase awareness of the benefits of, and promote recommended levels of, PA. International experiences could provide the much-needed inspiration. There is a need to design and implement country-, sociocultural-, and environmental-specific PA interventions. In addition, the effectiveness of policies and national interventions currently in place to promote PA in MENA countries must be continually assessed and evaluated to ensure that they are fulfilling the desired outcomes.

## Figures and Tables

**Table 1:** List of included systematic reviews with data on factors (barriers and facilitators) associated with physical activity in Middle East and North Africa (MENA) countries.

Systematics Review	Literature Search Period	Literature Search Geographical Coverage	Data Sources	MENA Countries with Identified Data	Number of Included Studies on MENA	Targeted Review Population
Mabry, 2016 [25]	Up to 2016	Oil-producing countries of the Arabian Peninsula	PubMed Web of Science Knowledge Google Scholar Web Search Engine	Saudi Arabia, UAE, Oman, Qatar, Kuwait, Bahrain	35	Youth and adult general population
Al-Hazzaa, 2018 [26]	Up to 15 January 2018	Saudi Arabia	MEDLINE and Google Scholar	Saudi Arabia	21	Youth and adult population
Sharara, 2018 [27]	2000 to 2016	22 Arab countries	MEDLINE, Popline and Social Sciences, Citation Index, reference lists of the articles, WHO surveys on non-communicable disease risk factors (STEPS), Global School-based Student Health Surveys (GSHS)	Algeria, Bahrain, Djibouti, Egypt, Iraq, Jordan, Kingdom of Saudi Arabia (KSA), Kuwait, Lebanon, Libya, Morocco, Oman, Palestine, Qatar, Sudan, Syria, Tunisia, UAE, and Yemen	79	Adult and children/adolescent
Benjamin 2013 [28]	2002–2013	Middle Eastern countries	MEDLINE, Cumulative Index to Nursing and Allied Health Literature (CINAHL), SPORTdiscus, and Middle Eastern and Central Asian Studies	Saudi Arabia, UAE, Kuwait, Qatar	7	Adult

**Table 2:** Factors (barriers and facilitators) associated with physical activity in MENA.

	Country	Direction of the Association	Factors Associated with Participation in PA
High income countries	Saudi Arabia	−	Age [29,30,31,32], [33] **, [34,35,36,37,38,39,40]
			Being a female [34,35,36,40,41]
			Being married [42] ^‡^, [29]
			Parity [43]
			High educational level [40]
			Less educated [42] ^‡^, [29,32,44,45,46,47]
			High occupational status [40,47]
			Employment (employed) [40,47]
			Self-efficacy [48], [49] ^†^, [50]
			Lack of motivation [31,40,50,51,52,53,54], [55] **, [42] ^‡^
			Lack of will-power [54]
			Lack of self-confidence [51]
			Perceived health [56], [42] ^‡^, [55] **, [50]
			Increased BMI and Waist circumference [29,36], [33] **,
			[57,58,59]
			Presence of diseases or health condition [30]
			Lack of time [60] **, [48], [45] ^†^, [50], [61] ^†^, [40,51], [42] ^‡^, [62] ^†^, [32,35,36,52,63,64,65], [55] **, [66]
			Working 7 h or more [45,62] ^†^
			Work in private sector [42] ^‡^, [62] ^†^
			Work duties [52]
			Shift duty [44]
			High screen time [41,50,53,58,67]
			Sleeping hours [36]
			Eating habits [36]
			Limited Knowledge/awareness of benefits of PA [34,66]
			Locus of control [51]
			Fear of criticism [56]
			Low value of PA [50]
			Enhance appearance [68]
			Social support (friends, parents, teachers) [45] ^†^, [39,40,41,50,51,55,64] **, [53,54,58]
			Lack of suitable sports facilities [60] **, [48], [45] ^†^, [50], [40,61] ^†^, [29,39,41,51,52,64,69,70], [48] **, [54], [49] ^†^, [35], [55] **, [40], [42] ^‡^
			No safe place [50]
			Urban residence [30,47]
			Inadequate school physical education program [57]
			Bad weather/ Hot climate [29,40,54,65,71,72,73]
			Gender & cultural norms (values and practices) [40,42,67]
		+	Age [74] ^‡^ [54]
			Being a male [29,30,32,35,36,40,75]
			Being married [44] ^†^ [29,76] ^‡^ [45] ^†^
			Employment (employed) [30]
			Self-efficacy [77]
			Stage of change [51]
			Increased BMI and Waist circumference [42,45] ^†^
			Lose/maintain weight [42] ^‡^ [45] ^†^ [61] ^†^ [49] ^†^ [60] ** [62] ^†^
			Health benefits [60] ** [42] ^‡^ [45] ^†^ [61] ^†^ [49] ^†^ [62] ^†^ [35]
			Consumption of foods high in fats/salt/sugar [35,36,74,75,78]
			Consumption of fruits [35,36,75]
			Consumption of vegetables [35,36]
			Consumption of milk [35,36]
			Transportation [74]
			Enhancing muscle and strength [55] **
			Enjoyment [55] ** [61] ^†^
			Improving body appearance [55] **
			Recreation [35] [60] ** [42] ^‡^ [49] ^†^
			Socializing [60] **, [35]
			Have fun [45] ^†^
	Kuwait	−	Age [79]
			Being married [80]
			Less educated [81]
			Perceived Health [82]
			Increased BMI and Waist circumference [80]
			Presence of diseases or health condition [82]
			Lack of time [7,82]
			Excessive use of private cars [82]
			Lack of exercise partner [82]
			Bad weather/ Hot climate [73,82,83]
			Gender & cultural norms (values and practices) [84]
			Having more domestic workers than one needs [82]
		+	Being married [84]
	UAE	−	Age [38,85,86]
			Being married [87]
			Less educated [86,87]
			Lack of motivation [63,88]
			Lack of will-power [88]
			Fear of injury [88]
			Exercise is boring [88]
			Embarrassed to wear exercise clothes [88]
			Belief that exercise makes control of diabetes difficult [88]
			Presence of diseases or health condition [88]
			Lack of time [88,89,90,91]
			Family responsibilities [88]
			High screen time [89]
			Limited Knowledge/ awareness of benefits of PA [92]
			Belief in overweight as normal [90]
			Attitude to changing diet [90]
			Low value of PA [90]
			Social support (friends, parents, teachers) [88]
			Lack of suitable sports facilities [63,88,89,90,93]
			No safe place [88,90]
			Cost of joining gym [88]
			Bad weather/ Hot climate [63,73,88,89,90,93,94]
			Limited material resources in health centers (teaching materials, guidelines) [90]
			Limited availability of human resources (i.e., dietitians) [90]
			Gender & cultural norms (values and practices) [38,63,89,93,94,95]
			Difficulty of joining gym for women-few centers for women only [88]
			Norms promoting overeating [90]
			Ineffective health communication [90]
			Ineffective PA supportive policies in colleges [63]
		+	Employment (employed) [87]
			Presence of diseases or health condition [89]
			Social support (friends, parents, teachers) [89]
			Living on a farm [89]
			Cooler weather [89]
	Qatar	−	Maintain health [68]
			Presence of diseases or health condition [96]
			Lack of time [68,97]
			Family responsibilities [96]
			Priority on caring for family-not exercise [96]
			High screen time [58,98]
			Limited Knowledge/ awareness of benefits of PA [68]
			Social support (friends, parents, teachers) [99]
			Lack of suitable sports facilities [58,99]
			Bad weather/ Hot climate [73,96]
			Gender & cultural norms (values and practices) [63,96]
			Taboo for females to go out in public places unless accompanied by male family member [96]
			Having more servants than one needs [96]
		+	Increased BMI and Waist circumference [97]
			Knowledge PA is important [99,100]
			Feeling healthy and looking younger participants expressed desire for slimmer bodies [99]
			Low cost and accessible facilities [99]
			Religion- Quran supportive of exercise [99]
	Bahrain	−	Lack of time [101]
			Bad weather/ Hot climate [73]
			Limited material resources in health centers (teaching materials, guidelines) [101]
			Lack of specialty clinics at primary health care level [101]
			Gender & cultural norms (values and practices) [13]
	Oman	−	Age [76] ^†^
			High educational level [76] ^‡^
			Lack of motivation [102,103]
			Increased BMI and Waist circumference [76]
			Lack of time [102,103]
			Consumption of fruits [76]
			Low value of PA [102]
			Social support (friends, parents, teachers) [102]
			Lack of suitable sports facilities [102]
			Bad weather/ Hot climate [73,102]
			Gender & cultural norms (values and practices) [102]
			Ineffective health communication [102]
		+	Age [76] ^‡^
			Employment (employed) [76]
			Consumption of vegetables [76]
Low- Middle income countries	Libya	−	Lack of suitable sports facilities [104]
	Tunisia	−	Age [85]
			Lack of time [105]
			Smoking [105,106]
			Urban residence [107]
			Gender & cultural norms, traditional cultural values and practices [105,108]
	Pakistan	−	Age [85]
	Lebanon	−	Age [109,110]
			Being married [110,111]
			High educational level [109]
			Less educated [112]
			Socioeconomic status [109]
			Increased BMI and Waist circumference [109,110,113]
			Presence of diseases or health condition [109,110]
			Lack of time [112]
			Alcohol [112]
			Smoking [110,112]
			Social support (friends, parents, teachers) [112]
			Lack of suitable sports facilities [114,115,116]
			Urban residence [109]
			Gender & cultural norms, traditional cultural values and practices [114]
		+	Employment (employed) [112,117]
			Socioeconomic status [111]
			Increased BMI and Waist circumference [112]
			Smoking [118]
	Morocco	−	Age [119]
			Being married [120]
			Socioeconomic status [120,121]
			Increased BMI and Waist circumference [119]
			Lack of suitable sports facilities [122,123]
			Gender & cultural norms, traditional cultural values and practices [121,122,123,124]
	Egypt	−	Less educated [125]
			Socioeconomic status [125]
			Lack of motivation [125,126]
			Presence of diseases or health condition [126]
			Lack of time [125]
			High screen time [127]
			Social support (friends, parents, teachers) [125,128]
			Lack of suitable sports facilities [125,128]
			Bad weather/Hot climate [125]
			Gender & cultural norms, traditional cultural values and practices [125,128]
	Syria	−	Age [129,130]
			Being married [129]
			Less educated [130]
			Socioeconomic status [130]
		+	Employment (employed) [130]
	Palestine	−	Age [131]
			Less educated [131]
			Urban residence [132]
			Gender & cultural norms, traditional cultural values and practices [132]
	Iraq	−	Age [133]
			Gender & cultural norms, traditional cultural values and practices [134]
	Jordan	−	Age [135]
			Gender & cultural norms, traditional cultural values and practices [136,137,138,139]
	Sudan	−	Age [140]
			Less educated [140]
Arab countries	Arab countries	−	Socioeconomic status [141]
			Lack of time [7]
			Social support (friends, parents, teachers) [7]
			Lack of suitable sports facilities [7,142]
			Bad weather/Hot climate [7]
			Gender & cultural norms, traditional cultural values and practices [7]

+: Factors positively associated with an increased participation in PA (facilitators). -: Factors negatively associated with an increased participation in PA (barriers). BMI: Body Mass Index. ^†^ Females only. ^‡^ Males only. ** Boys only.

## Supplementary Materials

The following are available online at https://www.mdpi.com/1660-4601/18/4/1647/s1, Panel 1: Search strategy.
